# Low pH effects on reactive oxygen species and methylglyoxal metabolisms in *Citrus* roots and leaves

**DOI:** 10.1186/s12870-019-2103-5

**Published:** 2019-11-06

**Authors:** An Long, Wei-Lin Huang, Yi-Ping Qi, Lin-Tong Yang, Ning-Wei Lai, Jiu-Xin Guo, Li-Song Chen

**Affiliations:** 1 Institute of Plant Nutritional Physiology and Molecular Biology, College of Resources and Environment, Fujian Agriculture and Forestry University, Fuzhou, 350002 China; 2 Institute of Materia Medica, Fujian Academy of Medical Sciences, Fuzhou, 350001 China; 3 Key Lab of Soil Ecosystem Health and Regulation, Fujian Province University, Fujian Agriculture and Forestry University, Fuzhou, 350002 China; 4 Key Lab of Soil Ecosystem Health and Regulation (Fujian Agriculture and Forestry University), Fujian Province University, Fuzhou, 350002 China

**Keywords:** Ascorbate metabolism, *Citrus*, Low pH, Methylglyoxal, Reactive oxygen species

## Abstract

**Background:**

Limited data are available on the responses of reactive oxygen species (ROS) and methylglyoxal (MG) metabolisms to low pH in roots and leaves. In China, quite a few of *Citrus* are cultivated in acidic soils (pH < 5.0). ‘Xuegan’ (*Citrus sinensis*) and ‘Sour pummelo’ (*Citrus grandis*) (*C. sinensis* were more tolerant to low pH than *C. grandis*) seedlings were irrigated daily with nutrient solution at a pH of 2.5, 3 or 5 for nine months. Thereafter, we examined low pH effects on growth, and superoxide anion production rate (SAP), malondialdehyde (MDA), MG, antioxidants, and enzymes related to ROS and MG detoxification in roots and leaves in order to (*a*) test the hypothesis that low pH affected ROS and MG metabolisms more in roots than those of leaves, and (*b*) understand the roles of ROS and MG metabolisms in *Citrus* low pH-tolerance and -toxicity.

**Results:**

Compared with control, most of the physiological parameters related to ROS and MG metabolisms were greatly altered at pH 2.5, but almost unaffected at pH 3. In addition to decreased root growth, many fibrous roots became rotten and died at pH 2.5. pH 2.5-induced changes in SAP, the levels of MDA, MG and antioxidants, and the activities of most enzymes related to ROS and MG metabolisms were greater in roots than those of leaves. Impairment of root ascorbate metabolism was the most serious, especially in *C. grandis* roots. pH 2.5-induced increases in MDA and MG levels in roots and leaves, decreases in the ratios of ascorbate/(ascorbate+dehydroascorbate) in roots and leaves and of reduced glutathione/(reduced+oxidized glutathione) in roots were greater in *C. grandis* than those in *C. sinensis*.

**Conclusions:**

Low pH affected MG and ROS metabolisms more in roots than those in leaves. The most seriously impaired ascorbate metabolism in roots was suggested to play a role in low pH-induced root death and growth inhibition. Low pH-treated *C. sinensis* roots and leaves had higher capacity to maintain a balance between ROS and MG production and their removal via detoxification systems than low pH-treated *C. grandis* ones, thus contribute to the higher acid-tolerance of *C. sinensis*.

## Background

About 30% of the world’s ice-free lands are acidic [1]. In China, acidic soils are observed in 15 provinces, comprising up to 21% of the arable lands [2]. Aluminum (Al)-toxicity and low pH (H^+^ rhizotoxicity) are two main factors limiting crop yield and quality in acidic soils [3, 4]. Considerable research has been performed to clarify the mechanisms of Al-toxicity and -tolerance in plants [5–11]. Nevertheless, limited data are available on the responses of plants to low pH [12–14]. Although some plants grow well in acidic soils with high level of active Al, the adaptive mechanisms of plants to low pH and Al-toxicity are not exactly the same [3, 15].

Overproduction and accumulation of reactive oxygen species (ROS) and methylglyoxal (MG) in plant cells in response to abiotic stresses is a common phenomenon [16–20]. ROS are scavenged by diverse enzymatic and non-enzymatic detoxification systems [21–23]. Antioxidant enzyme (thiol-based antioxidant) system has been regarded as the first (second) line of defense against the oxidative stress [24]. Sulfur (S) metabolism, a central pathway for the biosynthesis of S-containing compounds-namely reduced glutathione (GSH), cysteine (Cys) and H_2_S -plays important roles in plant tolerance to abiotic stresses [16, 25]. Mishra et al. demonstrated that thiol metabolism and antioxidant enzyme system complemented each other during the detoxification of arsenic in *Ceratophyllum demersum* plants [21]. The detoxification of MG is mainly undertaken by glyoxalase (Gly) I and Gly II. The coordinated actions of glyoxalases and antioxidant systems have been suggested to play a role in the alleviation of oxidative stress in plants [17].

A lot of evidence shows that efficient maintenance of redox homeostasis through detoxification systems of ROS and MG plays a role in Al-tolerance of higher plants [16, 26–28]. Nahar et al. [18] and Guo et al. [29] suggested that both spermidine-induced Al-tolerance of mung bean and S-induced Al-tolerance of *Citrus grandis* were associated with the enhanced ROS and MG detoxification systems. Additional evidence from transgenic plants indicates that increased activities of ROS scavenging enzymes through overexpressing tobacco glutathione S-transferase (GST), wheat manganese (Mn) superoxide dismutase (SOD), tobacco peroxidase, *Arabidopsis* cytosolic dehydroascorbate (DHA) reductase (DHAR) genes can mitigate Al-toxicity in transgenic *Arabidopsis* [30] canola [31] and tobacco [32] plants. However, it is unclear whether ROS and MG detoxification systems play a role in low pH (acid)-tolerance and -toxicity of higher plants or not.

Few studies investigated the antioxidant responses of roots and leaves (shoots) to low pH. The results are somewhat conflicting. Compared with pH 5.75-treated roots and shoots for 6 weeks, malondialdehyde (MDA) level was increased in pH 4.5-treated *Plantago algarbiensis* shoots and roots and pH 4.5-treated *Plantago almogravensis* roots, but not in pH 4.5-treated *P. almogravensis* shoots. SOD, catalase (CAT), ascorbate (ASC) peroxidase (APX) and guaiacol peroxidase (GuPX) activities did not change in response to pH. The exceptions were that the activities of APX in *P. algarbiensis* roots and of GuPX in *P. algarbiensis* leaves were higher at pH 4.5 than those at pH 5.75 [33]. However, pH 4 for seven days did not alter H_2_O_2_ and MDA levels, and CAT, APX, SOD and GuPX activities in *P. algarbiensis* and *P. almogravensis* leaves and roots relative to pH 5.75 [34]. Zhang et al. reported that pH 2.5-induced accumulation of H_2_O_2_ and MDA in rice roots was accompanied by decreased CAT and SOD activities, and increased APX activity [35]. Recently, we found that low pH decreased ASC concentration and ASC/(ASC + DHA) ratio in *C. grandis* and *C. sinensis* leaves, especially in the former, but had no influence on DHA concentration [12]. Unfortunately, reduced glutathione (GSH) and oxidized glutathione (GSSG) were not determined in all these studies. So far, very little is known about the responses of thiol metabolism, MG production and detoxification to low pH in higher plants.

In China, quite a few of *Citrus* are cultivated in acidic soils having a pH of less than 5.0. Furthermore, significant acidification is occurred in the major *Citrus* production areas in the last decade [36]. Although *Citrus* are insensitive (tolerant) to low pH [12, 14, 37], they can’t thrive in acidic soils having a pH of 5 or lower [38]. Indeed, *Citrus* often have poor growth and a shortened lifespan when grown in soils with a low pH and a high active Al [39]. In addition to greatly affecting Al-toxicity [40], low pH can directly impair root growth and function, thus interfering with water and nutrient uptake, and hence, inhibiting shoot growth of *Citrus* [14]. Field surveys revealed that low pH reduced yield and improved titratable acidity of *Citrus* fruits [41, 42]. Recently, we observed that both pH 2.5 and pH 3 increased H_2_O_2_ production rate (HP) in *C. sinensis* and *C. grandis* roots relative to pH 5, with a greater increase at pH 2.5, while only pH 2.5 increased HP in *C. sinensis* and *C. grandis* leaves and electrolyte leakage (EL) in *C. sinensis* and *C. grandis* roots and leaves, and that low pH-induced increases in HP and EL were greater in *C. grandis* roots and leaves than those in *C. sinensis* roots and leaves [14]. Thus, low pH-induced alterations of ROS and MG metabolism should be greater in roots than those in leaves of *Citrus* seedlings, and ROS and MG detoxification systems might have higher capacity to keep the redox homeostasis in *C. sinensis* roots and leaves than in *C. grandis* roots and leaves under low pH. Here, we examined low pH effects on growth, and superoxide anion production rate (SAP), MDA, MG, antioxidants, and enzymes related to ROS and MG detoxification in roots and leaves of *C. sinensis* and *C. grandis* seedlings differing in low pH-tolerance. The objectives were to (*a*) test the hypothesis that low pH affected ROS and MG metabolisms more in roots than those in leaves, and (*b*) understand the roles of ROS and MG metabolisms in *Citrus* low pH-tolerance and -toxicity.

## Methods

### Plant culture and pH treatments

Seedling culture and pH treatments were performed as described by Zhang et al. [12] and Long et al. [14]. Briefly, four week-old uniform seedlings of ‘Sour pummelo’ (*C. grandis*) and ‘Xuegan’ (*C. sinensis*) were chosen and transplanted to 6 L pots (two seedlings per pot) filled with ~ 0.5-cm-diameter sand washed thoroughly with tap water, then grown in a glasshouse under natural photoperiod at Fujian Agriculture and Forestry University, Fuzhou. *C. sinensis* were more tolerant to low pH than *C. grandis* [12, 14]. Seven weeks after transplanting, each pot was fertilized daily with freshly prepared nutrient solution until dripping (~ 500 mL) at a pH of 5 (control), 3 and 2.5 (adjusted by 1 M H_2_SO_4_ before supply). The nutrient solution was clear and transparent. The solubility of macroelements at various pH values was not affected [12]. pH was chosen according to the previous study in a pH range of 2.5–6, because only pH 2.5 greatly decreased seedling growth, pH 3 slightly decreased seedling growth, pH 4 hardly affected seedling growth, and seedling growth reached the optimum at pH 5 [14]. Nine months after the treatments began, the recent fully expanded (~ 7-week-old) leaves and ~ 5-mm-long white root apices were used for all measurements. White root apices and 6-mm-diameter leaf discs were harvested at sunny noon and frozen immediately in liquid N_2_, then stored at − 80 °C until extraction of enzymes and metabolites. These unsampled seedlings were used to determine SAP.

### SAP and metabolites in leaves and roots

SAP, GSH, GSSG, ASC and DHA concentrations were assayed according to Chen et al. [43]. MDA and MG concentrations were measured according to Hodges et al. [44] and Guo et al. [16], respectively.

### Enzyme activities in leaves and roots

Monodehydroascorbate (MDHA) reductase (MDHAR), APX, DHAR, glutathione reductase (GR), SOD, CAT, GuPX, glutathione peroxidase (GlPX) and GST were extracted according to Chen and Cheng [45]. GuPX and SOD activities were measured according to Chen et al. [46] and Giannopolitis and Ries [47], respectively. APX, MDHAR, GR, DHAR and CAT activities were measured as described previously [45]. GST and GlPX were determined as described by Guo et al. [16].

Ascorbate oxidase (AO) was extracted and assayed according to Pignocchi et al. [48]. Briefly, five leaf discs or ~ 60 mg roots were extracted in 1 mL extraction solution containing 50 mM potassium phosphate buffer (pH 7.0), 1 M KCl, 10 mM 2-mercaptoethanol, 1 mM phenylmethanesulfonyl fluoride (PMSF), and 4% (w/v) insoluble polyvinylpolypyrrolidone (PVPP). AO activity was measured following the decrease in A_265_ at 25 °C in 1 mL reaction solution containing 0.1 M sodium phosphate buffer (pH 5.6), 0.5 mM EDTA, 100 μM ASC, and 100 μL extract.

Phosphomannose isomerase (PMI) was extracted and measured according to Todd and Tague [49]. Briefly, six leaf discs or ~ 100 mg roots were extracted in 1 mL extraction solution containing 25 mM Tris-HC1 (pH 7.5), 5 mM dithiothreitol (DTT), 1 mM PMSF, and 4% (w/v) insoluble PVPP. PMI activity was assayed in 1 mM reaction solution containing 25 mM Tris-HC1 (pH 7.5), 5 mM MgC1_2_, 1 mM NADP, 1 U glucose-6-phosphate dehydrogenase, 0.5 U phosphoglucose isomerase, and 100 μL extract. The reaction was started with 3 mM mannose-6-phosphate (M6P).

ATP sulfurylase (ATPS), Cys synthase (CS), adenosine 5′-phosphosulphate (APS) reductase (APR), sulfite reductase (SiR), γ-glutamylcysteine synthetase (γGCS), Gly I and Gly II were extracted according to Cai et al. [23]. ATPS, CS, Sir and APR activities were assayed according to Guo et al. [29]. γGCS was assayed as described previously [23]. Gly I and Gly II were assayed according to Guo et al. [16].

Glutamine synthetase (GS) was extracted and assayed according to Li et al. [50].

### Statistical analysis

There were 20 pots per treatment in a completely randomized design. Experiments were performed with eight replicates except for SAP, MG, GR, SOD, CAT, GuPX, GST, GS, Gly I and Gly II (*n* = 4; one seedling from different pots per replicate). Significant differences among the six treatment combinations were analyzed by two (species) × three (pH levels) analysis of variance, and followed by the Duncan’s new multiple range test at *P* < 0.05.

Principal component analysis (PCA) and Pearson correlation analysis for all the measured physiological parameter were made with the SPSS statistical software (version 17.0, IBM, NY, USA) [51, 52].

## Results

### Low pH effects on seedling growth

Compared with pH 5, *C. grandis* and *C. sinensis* seedling growth was greatly reduced at pH 2.5, but displayed little changed at pH 3. pH 2.5-induced decreases in root and shoot DW were greater in *C. grandis* seedlings than those of *C. sinensis* seedlings. Although many fibrous roots became rotten and died, and the remaining living roots became abnormally dark brown in pH 2.5-treated seedlings, no seedlings died at each given pH until the end of this experiment. In addition, we observed mottled and/or bleached leaves in pH 2.5-treated *C. grandis* seedlings, and early shedding of the basal leaves in pH 2.5-treated *C. sinensis* seedlings (Additional file 1: Figure S1 and S2).

### Low pH effects on SAP, MDA and MG levels in leaves and roots

**Fig. 1:**
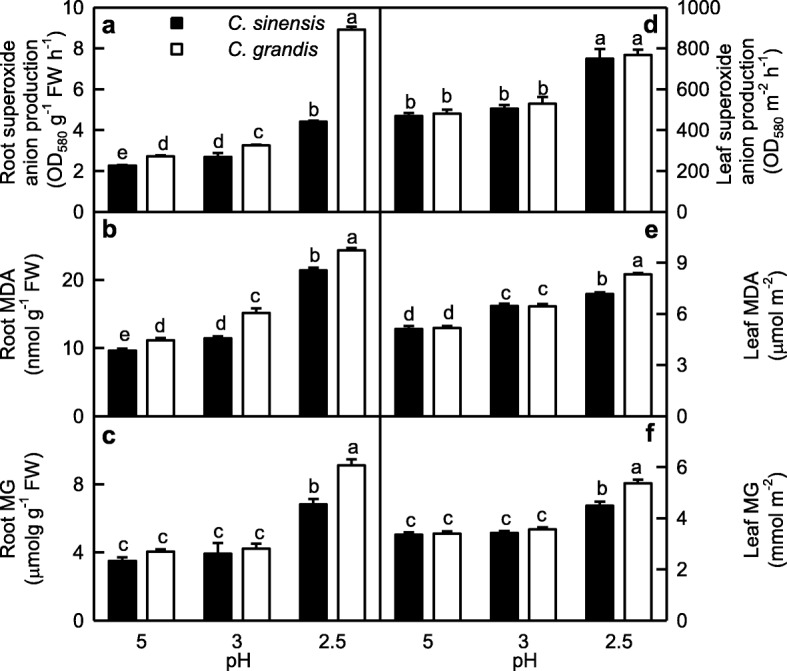
Effects of low pH on superoxide anion production rate (SAP; **a, d**), malondialdehyde (MDA; **b, e**) and methylglyoxal (MG; **c, f**) concentrations in *Citrus grandis* and *Citrus sinensis* roots (**a-c**) and leaves (**d-f**). Bar represent mean ± SE (*n* = 4 for superoxide anion production and MG or 8 for MDA). Different letters above the bars indicate a significant difference at *P* < 0.05

Root SAP increased as pH decreased from 5 to 2.5, while leaf ASP increased only at pH 2.5. SAP was higher in *C. grandis* roots than that of *C. sinensis* roots at each given pH, but it was similar between *C. grandis* and *C. sinensis* leaves (Fig. 1a, d).

MDA level in leaves and roots increased with decreasing pH. MDA level was similar between *C. grandis* and *C. sinensis* leaves at pH 3–5, but it was higher in *C. grandis* leaves than that of *C. sinensis* leaves at pH 2.5. MDA level was higher in *C. grandis* roots than that of *C. sinensis* roots at each given pH (Fig. 1b, e).

MG level in leaves and roots was higher at pH 2.5 than that at pH 3–5. MG concentration in roots and leaves was similar between the two *Citrus* species at pH 3–5, but its concentration was higher in *C. grandis* leaves than that in *C. sinensis* leaves at pH 2.5 (Fig. 1c, f).

### Low pH effects on the activities of enzymes involved in ROS and MG detoxification in leaves and roots

**Fig. 2:**
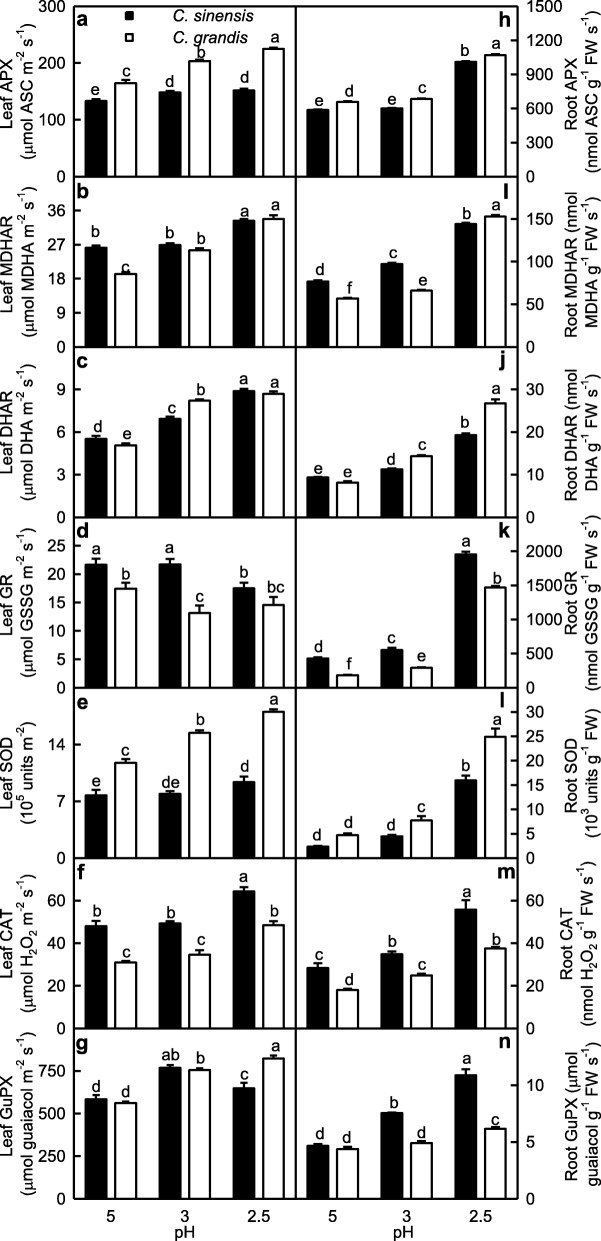
Effects of low pH on ascorbate peroxidase (APX; **a, h**), monodehydroascorbare reductase (MDHAR; **b, i**), dehydroascorbate reductase (DHAR; **c, j**), glutathione reductase (GR; **d, k**), superoxide dismutase (SOD, **e, l**), catalase (CAT, **f, m**) and guaiacol peroxidase (GuPX, **g, n**) activities in *Citrus grandis* and *Citrus sinensis* leaves (**a-g**) and roots (**h-n**). Bar represent mean ± SE (*n* = 4 except for 8 for APX, MDHAR and DHAR). Different letters above the bars indicate a significant difference at *P* < 0.05

We investigated low pH effects on the activities of antioxidant enzymes in leaves and roots (Fig. 2). APX, MDHAR, DHAR, SOD, CAT and GuPX activities in leaves decreased as pH increased from 2.5 to 3 except that GuPX activity in *C. sinensis* leaves was higher at pH 3 than that at pH 2.5, and that APX and SOD activities in *C. sinensis* leaves did not significantly change at pH 2.5–3, after which they remained stable or decreased with further increasing pH. GR activity in *C. sinensis* leaves was lower at pH 2.5 than that at pH 3–5, but its activity in *C. grandis* leaves remained stable at pH 2.5–5. APX, DHAR, SOD and GuPX activities were higher in *C. grandis* leaves than those in *C. sinensis* leaves or similar between the two at each given pH except that DHAR activity was higher in *C. sinensis* leaves than that in *C. grandis* leaves at pH 5. MDHAR, GR and CAT activities were higher in *C. sinensis* leaves than those in *C. grandis* leaves or similar between the two at each given pH (Fig. 2a-g).

The activities of the seven antioxidant enzymes in roots increased as pH decreased from 5 to 2.5 except that the activities of APX and SOD in *C. sinensis* roots and GlPX in *C. grandis* roots remained stable at pH 3–5. APX, DHAR and SOD activities were higher in *C. grandis* roots than those in *C. sinensis* roots or similar between the two at each given pH. MDHAR, GR, CAT and GuPX activities were higher in *C. sinensis* roots than those in *C. grandis* roots or similar between the two at each given pH except that MDHAR activity was higher in *C. grandis* roots than that in *C. sinensis* roots at pH 2.5 (Fig. 2h-n).

**Fig. 3:**
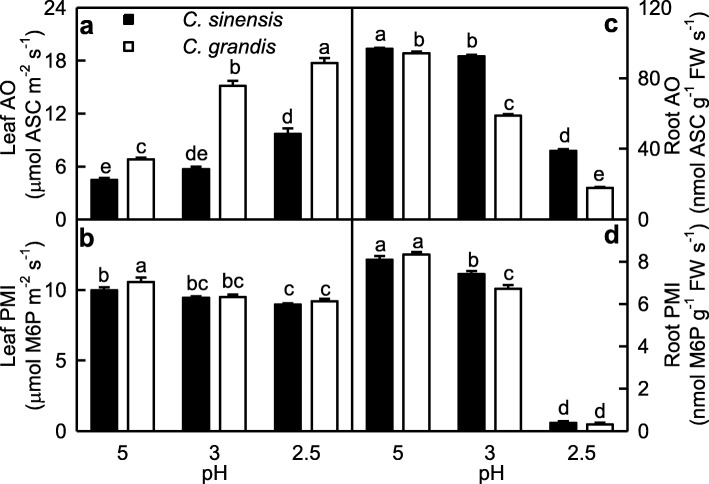
Effects of low pH on ascorbate oxidase (AO, **a, c**) and phosphomannose isomerase (PMI, **b, d**) activities in *Citrus grandis* and *Citrus sinensis* leaves (**a-b**) and roots (**c-d**). Bar represent mean ± SE (*n* = 8). Different letters above the bars indicate a significant difference at *P* < 0.05

We assayed the activities of OA involved in ASC oxidation and of PMI involved in ASC biosynthesis in roots and leaves (Fig. 3). Leaf AO activity increased with decreasing pH, while the reverse was the case for root AO activity. AO activity was higher in *C. grandis* leaves than that in *C. sinensis* leaves at each given pH, but its activity was higher in *C. sinensis* roots than that in *C. grandis* roots. PMI activity decreased with decreasing pH, with a greater decrease in roots than that in leaves. PMI activity was higher in *C. grandis* leaves than that in *C. sinensis* leaves or similar between the two at each given pH, but its activity was higher in *C. sinensis* roots than that in *C. grandis* roots or similar between the two.

**Fig. 4:**
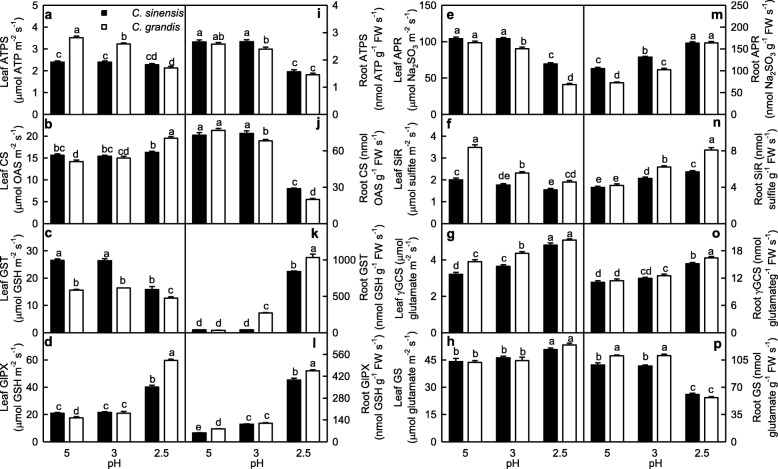
Effects of low pH on ATP sulphurylase (ATPS; **a, i**), cysteine synthase (CS; **b, j**), glutathione S-transferase (GST; **c, k**), glutathione peroxidase (GlPX; **d, l**), adenosine 5′-phosphosulfate reductase (APR; **e, m**), sulfite reductase (SiR; **f, n**), γ-glutamylcysteine synthase (γGCS; **g, o**) and glutamine synthetase (GS; **h, p**) activities in *Citrus grandis* and *Citrus sinensis* leaves (**a-h**) and roots (**i-p**). Bar represent mean ± SE (*n* = 8 except for 4 for GST and GS). Different letters above the bars indicate a significant difference at *P* < 0.05. OAS: O-acetyl-l-serine

Figure 4 displayed low pH effects on the activities of S metabolism-related enzymes in leaves and roots. ATPS activity in *C. grandis* leaves decreased with decreasing pH. CS activity in *C. grandis* leaves was higher at pH 2.5 than that at pH 3–5. However, ATPS and CS activities in *C. sinensis* leaves did not significantly change at pH 2.5–5. GST activity in *C. grandis* and *C. sinensis* leaves and APR activity in *C. sinensis* leaves were higher at pH 3–5 than those at pH 2.5, while APR activity in *C. grandis* leaves decreased with decreasing pH. GlPX and GS activities in *C. grandis* and *C. sinensis* leaves were higher at pH 2.5 than those at pH 3–5. SiR activity in *C. grandis* and *C. sinensis* leaves decreased with decreasing pH, while γGCS activity in *C. sinensis* and *C. grandis* leaves increased with decreasing pH. ATPS, CS, GlPX, SiR, γGCS and GS activities were higher in *C. grandis* leaves than those in *C. sinensis* leaves or similar between the two at each given pH except that CS and GlPX activities were lower in *C. grandis* leaves than those in *C. sinensis* leaves at pH 5. GST and APR activities were higher in *C. sinensis* leaves than those in *C. grandis* leaves or similar between the two at each given pH.

ATPS and GS activities in *C. grandis* and *C. sinensis* roots and CS activity in *C. sinensis* roots were higher at pH 3–5 than those at pH 2.5, while CS activity in *C. grandis* roots decreased with decreasing pH. GST, GlPX, APR, SiR and γGCS activities in *C. sinensis* and *C. grandis* roots increased with decreasing pH except that GST and γGCS activities in *C. sinensis* roots remained little changed at pH 3–5. ATPS, CS and APR activities were higher in *C. sinensis* roots than those in *C. grandis* roots or similar between the two at each given pH, while the reverse was the case for the activities of the other enzymes in roots.

**Fig. 5:**
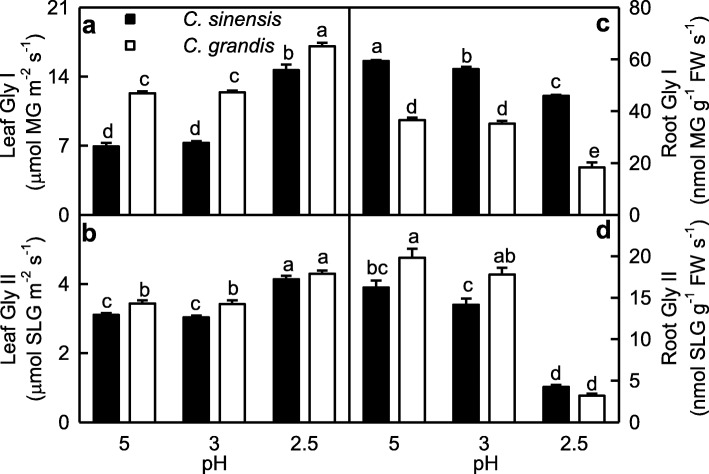
Effects of low pH on glyoxalase I (Gly I; **a, c**) and Gly II (**b, d**) activities in *Citrus grandis* and *Citrus sinensis* leaves (**a-b**) and roots (**c-d**). Bar represent mean ± SE (*n* = 4). Different letters above the bars indicate a significant difference at *P* < 0.05. SLG: S-D-lactoylglutathione

Gly I and Gly II activities in leaves were higher at pH 2.5 than those at pH 3–5, but their activities in roots were higher at pH 3–5 than those at pH 2.5. Gly I and Gly II activities were higher in *C. grandis* roots (leaves) than those in *C. sinensis* roots (leaves) or similar between the two at each given pH except that Gly I activity was higher in *C. sinensis* roots than that *C. grandis* roots (Fig. 5).

### Low pH effects on antioxidants in leaves and roots

**Fig. 6:**
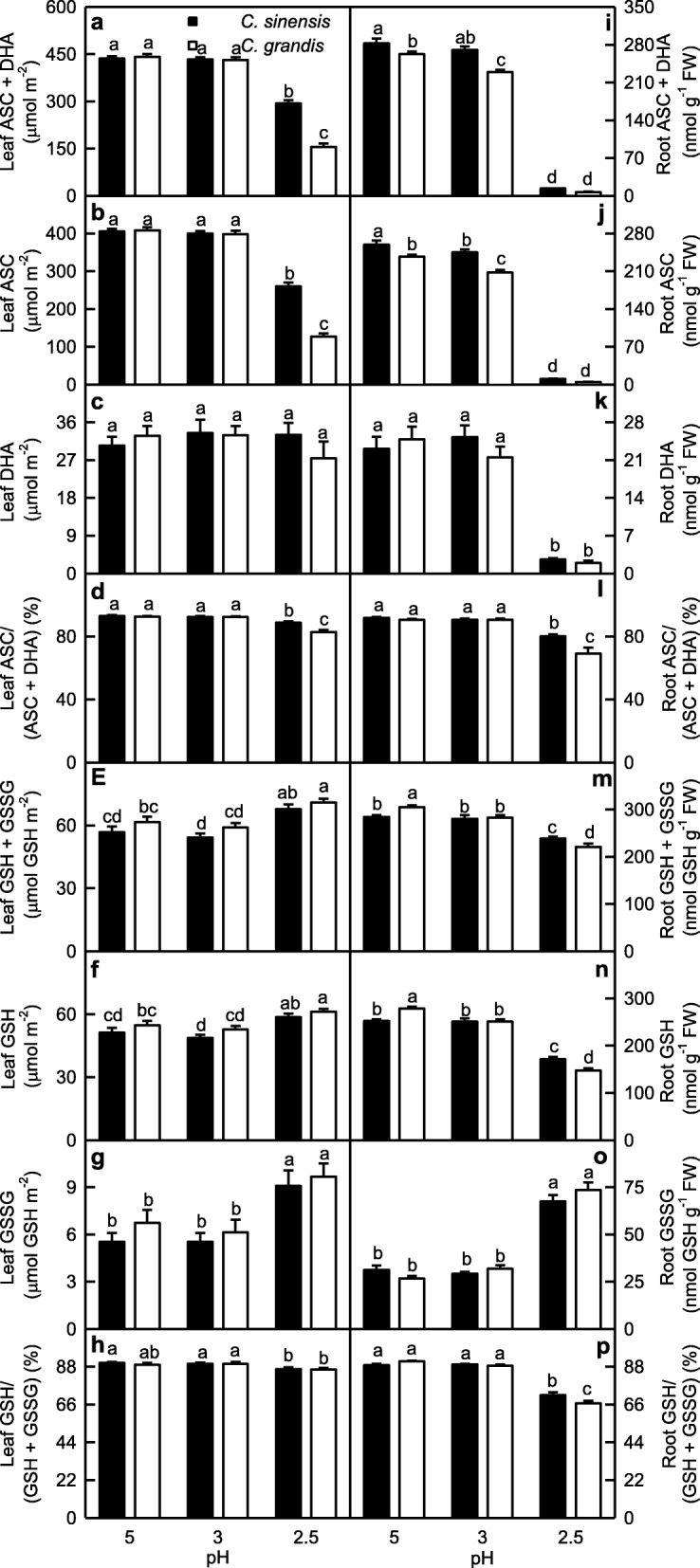
Effects of low pH on [ascorbate (ASC) + dehydroascorbate (DHA)] (**a, i**), ASC (**b, j**) and DHA (**c, k**) concentrations, ASC/(ASC + DHA) ratio (**d, l**), [reduced glutathione (GSH) + oxidized glutathione (GSSG)] (**e, m**), GSH (**f, n**) and GSSG (**g, o**) concentrations, and GSH/(GSH + GSSG) ratio (**h, p**) in *Citrus grandis* and *Citrus sinensis* leaves (**a-h**) and roots (**i-p**). Bar represent mean ± SE (*n* = 8). Different letters above the bars indicate a significant difference at *P* < 0.05

ASC and (ASC + DHA) levels and ASC/(ASC + DHA) ratio in *C. sinensis* and *C. grandis* leaves were higher at pH 3–5 that those at pH 2.5. The three parameters were higher in *C. sinensis* leaves than those in *C. grandis* leaves at pH 2.5, but they were similar between the two at pH 3–5. No significant difference was observed in leaf DHA level among the six treatment combinations (Fig. 6a-d).

ASC and (ASC + DHA) levels in roots decreased as pH decreased from 5 to 2.5 except that (ASC + DHA) level in *C. sinensis* roots did not significantly change at pH 3–5. DHA level and ASC/(ASC + DHA) ratio in roots were higher at pH 3–5 than those at pH 2.5. All the four parameters were higher in *C. sinensis* roots than those in *C. grandis* roots or similar between the two (Fig. 6i-l).

In leaves, (GSH + GSSG), GSH and GSSG concentrations kept unchanged as pH decreased from 5 to 3, then increased at pH 2.5. GSH/(GSH + GSSG) ratio did not change as pH decreased from 5 to 3, then slightly decreased at pH 2.5. All the four parameters did not significantly differ between *C. sinensis* and *C. grandis* leaves at each given pH (Fig. 6e-h).

GSH and (GSH + GSSG) concentrations in *C. sinensis* roots were higher at pH 3–5 than those at pH 2.5, while their concentrations in *C. grandis* roots decreased with decreasing pH. GSSG concentration in roots was higher at pH 2.5 than that at pH 3–5, but the reverse was the case for GSH/(GSH + GSSG) ratio. GSH and (GSH + GSSG) concentrations were higher in *C. grandis* roots than those in *C. sinensis* roots at pH 5, but GSH and (GSH + GSSG) concentrations and GSH/(GSH + GSSG) ratio were higher in *C. sinensis* roots than those in *C. grandis* roots at pH 2.5 (Fig. 6m-p).

### Relationships between parameters

In roots, AO activity was positively related to ASC level, DHA level or ASC/(ASC + DHA) ratio; PMI activity was positively related to ASC level or ASC/(ASC + DHA) ratio. In leaves, no such significant relationships were observed (Additional file 1: Figure S3).

In roots, MG level was negatively related to GSH level or Gly II activity, but only displayed a decreased trend with increasing Gly I activity. In leaves, MG level was positively related to GSH level, Gly I or Gly II activity (Additional file 1: Figure S4).

### PCA loading plots and Pearson correlation coefficient matrices

**Fig. 7:**
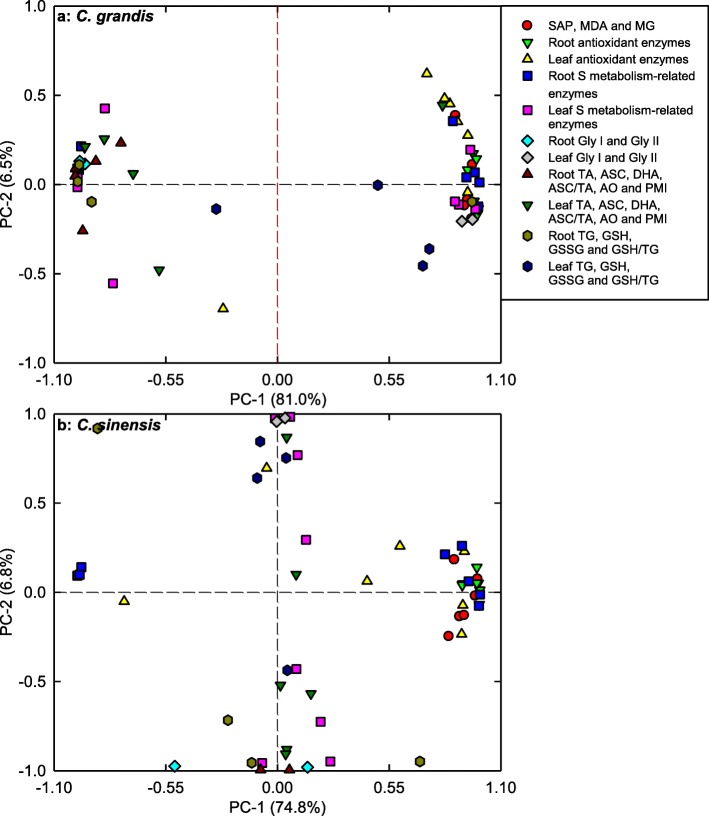
Principal component analysis (PCA) loading plots of physiological parameters of *Citrus grandis* (**a**) and *Citrus sinensis* (**b**) seedlings exposed to different pH levels. TA: ascrobate (ASC) + dehydroascorbate (DHA); TG: reduced glutathione (GSH) + oxidized glutathione (GSSG)

We observed that PC1 and PC2 contributed 81.0 and 6.5%, and 74.8 and 6.8% of the total variation in *C. grandis* and *C. sinensis* seedlings, respectively (Fig. 7; Additional file 1: Tables S1; S2), indicating that these physiological parameters were highly separated in different pH-treated *C. grandis* and *C. sinensis* seedlings, especially in the former. In *C. grandis* seedlings, root PMI (− 0.9963), root GST (0.9954), root ASC (− 0.9935), root (ASC + DHA) (− 0.9922) and root GlPX (0.9890) contributed largely to PC1. In *C. sinensis* seedlings, PC1 was loaded heavily on root GlPX (0.9970), root GR (0.9963), root ASC (− 0.9952), root (ASC + DHA) (− 0.9945) and root APX (0.9939).

**Fig. 8:**
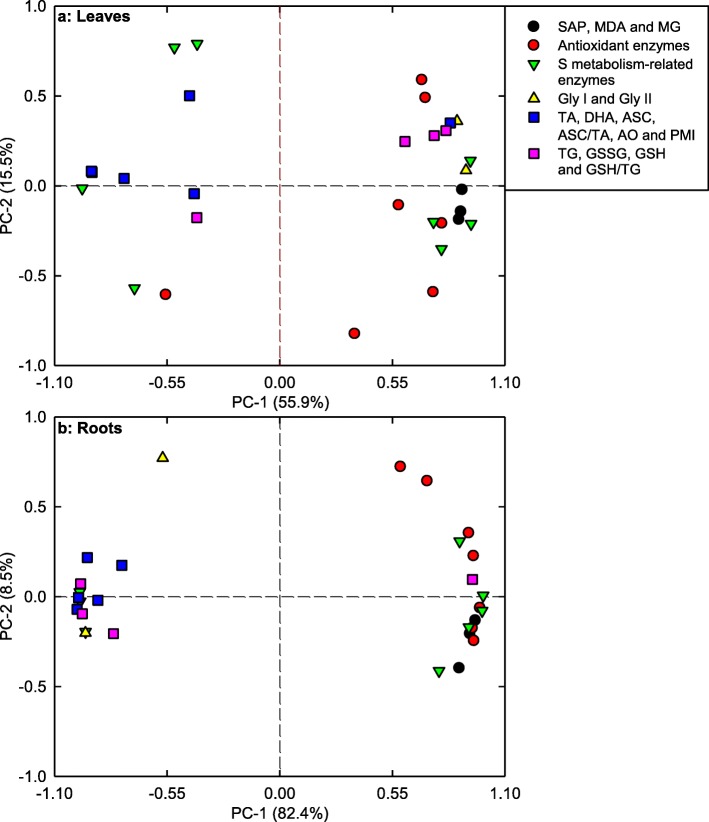
Principal component analysis (PCA) loading pots of physiological parameters of leaves (**a**) and roots (**b**) from *Citrus grandis* and *Citrus sinensis* seedlings exposed to different pH levels. TA: ascrobate (ASC) + dehydroascorbate (DHA); TG: reduced glutathione (GSH) + oxidized glutathione (GSSG)

PC1 and PC2 accounted for 55.9 and 15.5%, and 82.4 and 8.5% of the total variation in leaves and roots, respectively. Theses physiological parameters were highly clustered into two groups in roots, but not in leaves (Fig. 8; Additional file 1: Table S3). In leaves, PC1 was heavily loaded on APR (− 0.9647), GlPX (0.9346), γGCS (0.9315), ASC + DHA (− 0.9170) and ASC (− 0.9192). In roots, GlPX (0.9933), PMI (− 0.9893), GST (0.9881), ASC (− 0.9830) and AO (− 0.9402) were the main contributors for PC1 (Additional file 1: Table S3).

Most of the physiological parameters were positively or negatively related with each other in *C. grandis* and *C. sinensis* seedlings, especially in the former (Additional file 1: Figure S5). Majority of the physiological parameters were related with each other in roots, but not in leaves (Additional file 1: Figure S6).

## Discussion

### Low pH affected ROS and MG metabolisms more in roots than those in leaves

SAP in roots increased as pH decreased from 5 to 2.5, while only pH 2.5 increased SAP in leaves (Fig. 1a-b). This agrees with low pH-induced increase in HP in *C. sinensis* and *C. grandis* leaves and roots [14]. Although the change patterns of MDA and MG levels in response to pH were similar between roots and leaves, pH 2.5-induced increases in MDA and MG levels were greater in roots than those in leaves (Fig. 1). Similarly, pH 2.5-induced alterations of (ASC + DHA), ASC, DHA, (GSH + GSSG), GSH and GSSG levels, and ASC/(ASC + DHA) and GSH/(GSH + GSSG) ratios were greater in roots than those in leaves. Also, pH 2.5-induced changes in the activities of most enzymes related to ROS and MG detoxification were greater in roots than those in leaves (Figs. 2-6). PCA showed that all the physiological parameters were highly clustered into two groups in roots, but not leaves (Fig. 8). Pearson correlation analysis indicated that most of the physiological parameters were positively or negatively related with each other in roots, but not in leaves (Additional file 1: Figure S6). Obviously, low pH effects on ROS and MG metabolisms were greater in roots than those in leaves. This agrees with our results that many fibrous roots became rotten and died in pH 2.5-treated *C. sinensis* and *C. grandis* seedlings, but only mottled and/or bleached leaves and early shedding of the basal leaves occurred in some pH 2.5-treated *C. grandis* and *C. sinensis* seedlings, respectively (Additional file 1: Figure S1), and the report that low pH could directly impair *Citrus* root growth and function, thus interfering with the uptake of mineral nutrients and water, and affecting shoot growth [14].

**Fig. 9:**
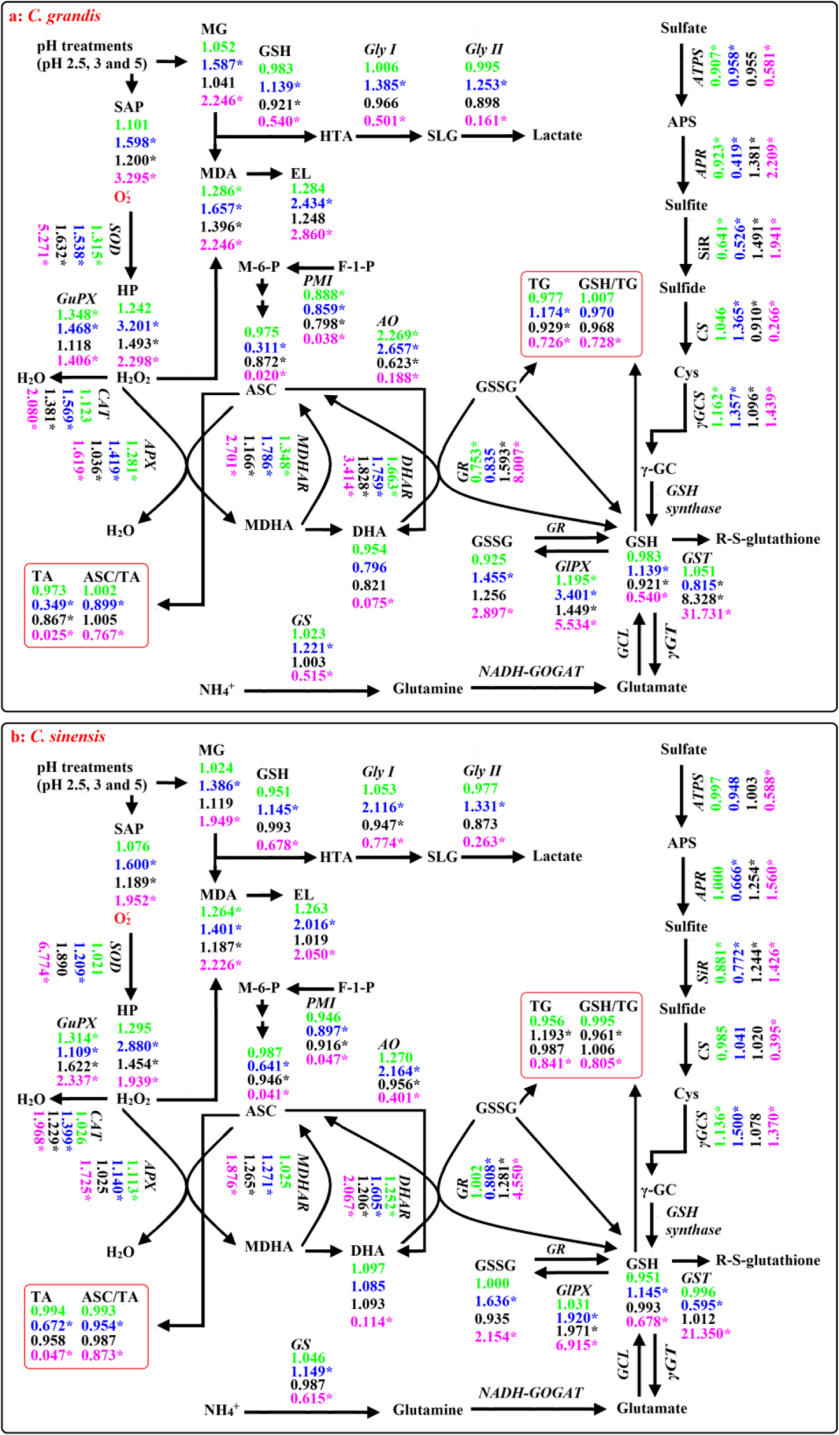
A diagram showing low pH effects on ROS and MG metabolisms in *C. grandis* (**a**) and *C. sinensis* (**b**) leaves and roots. In this Figure, we used italics for enzymes and plain format for metabolites. Data from Figs. 1-6 except for H_2_O_2_ production rate (HP) and electrolyte leakage (EL) from reference [14]. Values in green and blue (black and magenta) were the ratios of pH 3 and pH 2.5 to pH 5 in leaves (roots), respectively. An asterisk indicates a significant difference between pH 2.5 (pH 3) and pH 5 at *P* < 0.05. An enzyme or metabolite was considered increased or decreased when it had both a relative change of more or less, respectively, than 1 and a *P*-value of < 0.05. Metabolite concentrations and enzyme activities were determined on a whole tissue extract and not on a subcellular level. GCL: Glutamate-cysteine ligase; NADH-GOGAT: NADH-dependent glutamine-2-oxoglutarate aminotransferase; γGC: γ-glutamylcysteine; γGT: γ-glutamyltransferase; TA: Ascorbate (ASC) + dehydroascorbate (DHA); TG: Reduced GSH + GSSG

Except for GR activity in leaves, the activities of all the seven antioxidant enzymes were increased in pH 2.5-treated roots and leaves, with a greater increase in roots (Fig. 2). The higher upregulation of antioxidant enzymes in pH 2.5-treated roots than that in pH 2.5-treated leaves agrees with the increased requirement for ROS scavenging, because pH 2.5-induced increase in SAP was higher in roots than that in leaves. However, the upregulation of the antioxidant enzymes as a whole did not provide sufficient protection to them against the oxidative damage, because pH 2.5 increased MDA level and EL in roots and leaves, especially in roots (Figs. 1 and 9) [14]. We found that pH 2.5-induced decreases in the ASC/(ASC + DHA) [12] and GSH/(GSH + GSSG) ratios were greater in roots than those in leaves (Fig. 6). This also supported the above inference that pH 2.5-induced oxidative damage was more serious in roots than that in leaves, because ASC/(ASC + DHA) and GSH/(GSH + GSSG) ratios are reduced by oxidative stress [16, 53].

Thiol-based antioxidant system is the second line of defense against the oxidative stress. ATPS, which catalyzes the first reaction in the assimilation of inorganic sulfate and yields APS, is considered to be a rate-limiting enzyme. APS can be reduced to sulfide by the two sequential reactions catalyzed by APR and SiR, respectively [25]. Khan et al. showed that cadmium-induced increases in ATPS activity, and Cys and GSH production were higher in high photosynthetic potential *Brassica juncea* cultivar than those in low photosynthetic cultivar, thus decreasing the oxidative stress of the former [54]. Here, pH 2.5-induced decrease of ATPS activity was less in leaves than that in roots (Fig. 4a, i). This agrees with the result that pH 2.5-induced oxidative stress was more serious in roots than that in leaves (Fig. 1). In a given plant cells, GSH level is determined by GSH biosynthesis, utilization and degradation. Sulfide can be incorporated into Cys, which is catalyzed by CS. Cys in turn serves as a precursor for the biosynthesis of GSH and phytochelatin peptides in response to oxidative stresses. The availability of Cys is a key factor for the biosynthesis of GSH [55]. GSH, which is biosynthesized from Cys, is catalyzed by γGCS, a rate-limiting enzyme and glutathione synthetase [56]. In plant cells, GS is also involved in the synthesis of GSH [57]. GSTs can catalyze the conversion of H_2_O_2_ using GSH as co-substrate, thereby yielding GSSG. The main reaction that GlPX catalyzes is: H_2_O_2_ + 2 GSH → 2 H_2_O + GSSG [58]. GR catalyzes the reduction of GSSG to GSH [59]. Here, pH 2.5-induced decrease in root GSH level was caused mainly by the decreased biosynthesis due to the decreased CS and GS activities and the increased utilization due to the increased GST and GlPX activities, while pH 2.5-induced increase in leaf GSH level was caused mainly by the increased biosynthesis due to the increased GS, CS and γGCS activities (Fig. 4). In roots, pH 2.5-induced increase in GSSG level was caused mainly by the increased formation of GSSG due to the increased GlPX and GST activities, while pH 2.5-induced decrease in GSH/(GSH + GSSG) ratio was caused by the increased production of GSSG and the decreased biosynthesis of GSH. In leaves, pH 2.5-induced increase in GSSG level and decrease in GSH/(GSH + GSSG) ratio were caused mainly by the increased formation of GSSG due to the increased GlPX activity (Fig. 4). This was also supported by the PCA showing that GlPX was the second largest contributor for leaf PC1, and that GlPX and GST were the first and third largest contributors for root PC1 (Additional file 1: Table S3). Thus, it is reasonable to assume that GlPX was a determinant for both GSSG level and GSSG/(GSH + GSSG) ratio in *Citrus* roots and leaves.

MG is mainly detoxified by Gly I and Gly II, where GSH acts as a cofactor [17]. Here, we found that GSH level (Fig. 6n), and Gly I and Gly II activities (Fig. 5) in roots were lower at pH 2.5 than those at pH 5, and that MG level was negatively related to GSH level, Gly I or Gly II activity in roots (Additional file 1: Figure S4), indicating that MG detoxification system was impaired in these roots. Thus, pH 2.5-induced accumulation of MG in roots (Fig. 1c) was caused by pH 2.5-induced production and the downregulated detoxification system of MG. However, pH 2.5-induced accumulation of MG in leaves was caused mainly by pH 2.5-stimulated production of MG, because MG detoxification system was upregulated in these leaves (Figs. 1f and 5a-b). The different responses of the MG detoxification system to low pH between leaves and roots could explain why pH 2.5-induced increase in MG level was greater in roots than that in leaves.

To conclude, low pH affected ROS and MG metabolisms more in roots than those of leaves (Fig. 9). This was also supported by PCA showing that all the first five main contributors for *C. sinensis* PC1 (root GlPX, root GR, root ASC, root ASC + DHA and root APX) and *C. grandis* (root PMI, root GST, root ASC, root ASC + DHA and root GlPX) seedlings were root physiological parameters (Additional file 1: Tables S1, S2), and that all the physiological parameters were highly clustered in left and right groups in roots, but not in leaves (Fig. 8), and by Pearson correlation analysis showing that most of the physiological parameters were negatively or positively each other in roots, but not in leaves (Additional file 1: Figure S6).

### *C. sinensis* roots and leaves had higher capacity to maintain a balance between the production and removal of ROS and MG than that of *C. grandis* ones at low pH

pH 2.5-induced increases in SAP and MDA and MG levels were greater in *C. grandis* roots and leaves than those in *C. sinensis* ones (Fig. 1). Likewise, pH 2.5-induced changes in the levels of (ASC + DHA) and ASC in roots and leaves and of (GSH + GSSG) and GSH in roots, and the ratios of ASC/(ASC + DHA) in roots and leaves and of GSH/(GSH + GSSG) in roots were greater in *C. grandis* seedlings than those in *C. sinensis* ones (Fig. 6). Also, pH 2.5-induced changes in the activities of quite a little of enzymes in leaves and roots were greater in *C. grandis* seedlings than those in *C. sinensis* seedlings (Fig. 2-5). PCA showed that low pH-induced alterations of all the physiological parameters as a whole were greater in *C. grandis* seedlings than those in *C. sinensis* seedlings (Fig. 7). Pearson correlation analysis indicated that most of the physiological parameters were positively or negatively related with each other in *C. grandis* and *C. sinensis* seedlings, especially in the former (Additional file 1: Figure S5). Obviously, low pH effects on ROS and MG metabolisms were greater in *C. grandis* roots and leaves than those in *C. sinensis* roots and leaves (Fig. 9).

As shown in Figs. 2 and 9, except for unaltered (decreased) GR activity in *C. grandis* (*C. sinensis*) leaves, the activities of all the seven antioxidant enzymes were increased in pH 2.5-treated *C. grandis* and *C. sinensis* roots and leaves. pH 2.5-induced increases in antioxidant enzyme activities were greater in *C. grandis* roots and leaves than those in *C. sinensis* ones with few exceptions in order to cope with the increased requirement of ROS scavenging, as indicated by the greater increases in SAP and HP in pH 2.5-treated *C. grandis* roots and leaves than those of pH 2.5-treated *C. sinensis* roots and leaves except for a similar increase in leaf SAP between the two *Citrus* species (Figs. 1d and 9) [14]. The enhancement of antioxidant enzymes as a whole did not protect them from oxidative damage, especially in pH 2.5-treated *C. grandis* roots and leaves, as indicated by the greater MDA level and EL, and the lower ratios of ASC/(ASC + DHA) and GSH/(GSH + GSSG) in pH 2.5-treated *C. grandis* roots and leaves than those of pH 2.5-treated *C. sinensis* roots and leaves except for a similar leaf ratio of GSH/(GSH + GSSG) between the two *Citrus* species (Fig. 6) [14].

We found that ATPS activity was decreased in pH 2.5-treated *C. grandis* leaves, but not in pH 2.5-treated *C. sinensis* leaves (Fig. 4a), which agrees with the result that pH 2.5-induced oxidative stress was more serious in *C. grandis* leaves than that of *C. sinensis* leaves (Fig. 1). However, pH 2.5-induced decrease in ATPS activity was not greater in *C. grandis* roots than that of *C. sinensis* roots (Fig. 4i). In this study, the greater decrease in the ratio of GSH/(GSH + GSSG) in pH 2.5-treated *C. grandis* roots than that of pH 2.5-treated *C. sinensis* roots (Fig. 6p) was caused by the more increased production of GSSG due to the more increase in GST activity and higher GlPX activity and the more decreased biosynthesis of GSH due to the more decrease in GS and GS activities in former (Figs. 4 and 9).

As shown in Figs. 5 and 9, pH 2.5-induced increases in Gly I and Gly II activities were less in *C. grandis* leaves than those of *C. sinensis* leaves, while pH 2.5-induced decreases in Gly I and Gly II activities were greater in *C. grandis* roots than those of *C. sinensis* roots. This could explain why pH 2.5-induced increase in MG level was greater in *C. grandis* roots and leaves than that of *C. sinensis* roots and leaves.

To conclude, pH 2.5-treated *C. sinensis* roots and leaves had higher capacity to maintain a balance between the production of ROS and MG and their removal via detoxification systems than that of pH 2.5-treated *C. grandis* roots and leaves, thus protecting them against the oxidative damage. This agrees with the results that pH 2.5-induced declines in root and shoot DW were greater in *C. grandis* seedlings than those in *C. sinensis* seedlings (Additional file 1: Figure S2), and the report that *C. sinensis* were more tolerant to low pH than *C. grandis* [14].

### Impaired ASC metabolism played a role in low pH-induced death and growth inhibition of roots

In addition to protecting plant cells against the oxidative stress, evidence shows that ASC play a key role in plant cell division and growth [60, 61]. Lukaszewski and Blevins demonstrated that root growth inhibition in Al-toxic or boron-deficient squash was caused by impaired ASC metabolism [62]. One role for ASC in plant growth is that ASC can acts as a cofactor in the biosynthesis of cell wall structural proteins: hydroxyproline-rich glycoproteins [60]. In the “Smirnoff-Wheele” pathway (biosynthesis of ascorbic acid in plants via D-mannose and L-galactose), ASC is synthesized from GDP-mannose. GDP-mannose can be synthesized from fructose-6-phosphate by three sequential reactions catalyzed by the three enzymes: PMI, phosphomannose mutase and GDP-mannose pyrophosphorylase [63]. Maruta et al. reported that phosphomannose isomerase 1 (PMI1) was required for the biosynthesis of ASC [64]. The “Smirnoff-Wheele” pathway shares GDP-sugar intermediates with the biosynthesis of cell wall glycoproteins containing mannose, fucose and galactose and of cell wall polysaccharides [63]. Pignocchi et al. showed that increased activity of AO, a cell wall-bound enzyme, oxidized apoplastic ASC pool, while decreased activity of AO enhanced the relative level of ASC to DHA, and that there was a close relationship between the activity of AO and the height and biomass of *Arabidopsis* plants [48]. As shown in Figs. 3 and 6, pH 2.5-induced decreases in (ASC + DHA), ASC and DHA levels, ASC/(ASC + DHA) ratio and PMI activity were far greater in roots than those in leaves. Indeed, (ASC + DHA), ASC and DHA levels and PMI activity were very low in pH 2.5-treated roots. In addition, root AO activity was greatly decreased at pH 2.5, but the reverse was the case for leaf AO activity. In roots, PMI activity increased with increasing ASC level or ASC/(ASC + DHA) ratio; AO activity increased with increasing ASC level, DHA level or ASC/(ASC + DHA) ratio. In leaves, PMI (AO) activity displayed an increased (a decreased) trend with increasing ASC level or ASC/(ASC + DHA) ratio (Additional file 1: Figure S3). Decreased ASC level and ASC/(ASC + DHA) ratio in pH 2.5-treated roots were mainly caused by the decreased ASC biosynthesis due to the decreased PMI activity. Decreased ASC level and ASC/(ASC + DHA) ratio in pH 2.5-treated leaves, however, resulted from the increased oxidation due to the increased AO activity, and the decreased biosynthesis due to the decreased PMI activity (Fig. 3). Obviously, ASC metabolism was greatly impaired in pH 2.5-treated roots, but less in pH 2.5-treated leaves. Therefore, we concluded that impaired ASC metabolism played a role in low pH-induced death and growth inhibition of roots. This was also supported by PCA showing that PMI, ASC and AO were the second, fourth and fifth main contributors for root PC1 (Additional file 1: Table S3). It is worth noting that pH 2.5-induced impairment of ASC metabolism was less serious in *C. sinensis* roots and leaves than that in *C. grandis* ones (Figs. 3 and 6), thus enhancing the acid-tolerance of *C. sinensis*.

## Conclusions

We found that most of these parameters were altered greatly at pH 2.5, but almost unaffected at pH 3, suggesting that both *C. sinensis* and *C. grandis* seedlings were tolerant to low pH. Our findings clearly demonstrated that low pH affected ROS and MG metabolisms more in roots than those in leaves. Among them, low pH-induced impairment of ASC metabolism in roots was the most serious, with a greater impairment in *C. grandis* roots than that of *C. sinensis* roots. Low pH-treated *C. sinensis* roots and leaves had higher capacity to maintain a balance between the production of ROS and MG and their removal via detoxification systems than that of low pH-treated *C. grandis* ones, thus protecting them against the oxidative damage. Our findings suggested that MG and ROS were involved in the low pH (acid)-tolerance of *Citrus*, and that impaired ASC metabolism in low pH-treated roots played a role in low pH-induced root death and growth inhibition.

## Supplementary information

**Additional file 1: Figure S1.** Effects of low pH on *Citrus sinensis* (**a**) and *Citrus grandis* (**b**) seedlings, *C. grandis* leaves (**c**), and *C. sinensis* (**d**) and *C. grandis* (**e**) roots. **Figure S2.** Effects of low pH on root (**a**) and shoot (**b**) dry weight (DW) of *Citrus grandis* and *Citrus sinensis* seedlings. **Figure S3.** Phosphomannose isomerase (PMI) and ascorbate (ASC) oxidase (AO) activities in relation to ASC and dehydroascorbate (DHA) concentrations and ASC/(ASC + DHA) ratio in leaves (**a-e**) and roots (**f-j**). **Figure S4.** Reduced glutathione (GSH) concentration and glyoxalase (Gly) I and Gly II activities in relation to methylglyoxal (MG) concentration in leaves (**a-c**) and roots (**d-f**). **Figure S5.** Matrices of Pearson correlation coefficients among the 60 physiological parameters in *Citrus grandis* (**a**) and *Citrus sinensis* (**b**) seedlings. **Figure S6.** Matrices of Pearson correlation coefficients among the 30 physiological parameters in leaves (a) and roots (**b**). **Table S1.** Principal component analysis (PCA) for physiological parameters of *Citrus grandis* seedlings. **Table S2.** Principal component analysis (PCA) for physiological parameters of *Citrus sinensis* seedlings**. Table S3.** Principal component analysis (PCA) for physiological parameters of leaves and roots.

## Data Availability

All data analyzed in this study are included in this published article and its additional files.

## Abbreviations

Al: Aluminum
AO: Ascorbate oxidase
APR: Adenosine 5′-phosphosulfate reductase
APS: Adenosine 5′-phosphosulfate
APX: Ascorbate peroxidase
ASC: Ascorbate
ATPS: ATP sulphurylase
CAT: Catalase
CS: Cysteine synthase
Cys: Cysteine
DHA: Dehydroascorbate
DHAR: DHA reductase
DTT: Dithiothreitol
EL: Electrolyte leakage
GlPX: Glutathione peroxidase
Gly I: Glyoxalase I
Gly II: Glyoxalase II
GR: Glutathione reductase
GS: Glutamine synthetase
GSH: Reduced glutathione
GSSG: Oxidized glutathione
GST: Glutathione S-transferase
GuPX: Guaiacol peroxidase
HP: H_2_O_2_ production rate
M6P: Mannose-6-phosphate
MDA: Malondialdehyde
MDHA: Monodehydroascorbate
MDHAR: MDHA reductase
MG: Methylglyoxal
OAS: O-acetyl-l-serine
PMI: Phosphomannose isomerase
PMSF: Phenylmethanesulfonyl fluoride
PVPP: Polyvinylpolypyrrolidone
ROS: Reactive oxygen species
SAP: Superoxide anion production rate
SiR: Sulfite reductase
SLG: S-D-lactoylglutathione
SOD: Superoxide dismutase.
TA: ASC + DHA.
γGCS: γ-glutamylcysteine synthase
γGT: γ-glutamyltransferase
