# Supplementary material for: Low pH effects on reactive oxygen species and methylglyoxal metabolisms in Citrus roots and leaves
Source: BMC Plant Biol. 2019 Nov 6;19:477. doi: 10.1186/s12870-019-2103-5 (PMC6836343; doi:10.1186/s12870-019-2103-5)
Supplement: Supplementary file 1 — Additional file 1: Figure S1. Effects of low pH on Citrus sinensis (a) and Citrus grandis (b) seedlings, C. grandis leaves (c), and C. sinensis (d) and C. grandis (e) roots. Figure S2. Effects of low pH on root (a) and shoot (b) dry weight (DW) of Citrus grandis and Citrus sinensis seedlings. Figure S3. Phosphomannose isomerase (PMI) and ascorbate (ASC) oxidase (AO) activities in relation to ASC and dehydroascorbate (DHA) concentrations and ASC/(ASC + DHA) ratio in leaves (a-e) and roots (f-j). Figure S4. Reduced glutathione (GSH) concentration and glyoxalase (Gly) I and Gly II activities in relation to methylglyoxal (MG) concentration in leaves (a-c) and roots (d-f). Figure S5. Matrices of Pearson correlation coefficients among the 60 physiological parameters in Citrus grandis (a) and Citrus sinensis (b) seedlings. Figure S6. Matrices of Pearson correlation coefficients among the 30 physiological parameters in leaves (a) and roots (b). Table S1. Principal component analysis (PCA) for physiological parameters of Citrus grandis seedlings. Table S2. Principal component analysis (PCA) for physiological parameters of Citrus sinensis seedlings. Table S3. Principal component analysis (PCA) for physiological parameters of leaves and roots. [file 12870_2019_2103_MOESM1_ESM.pdf]

Additional files

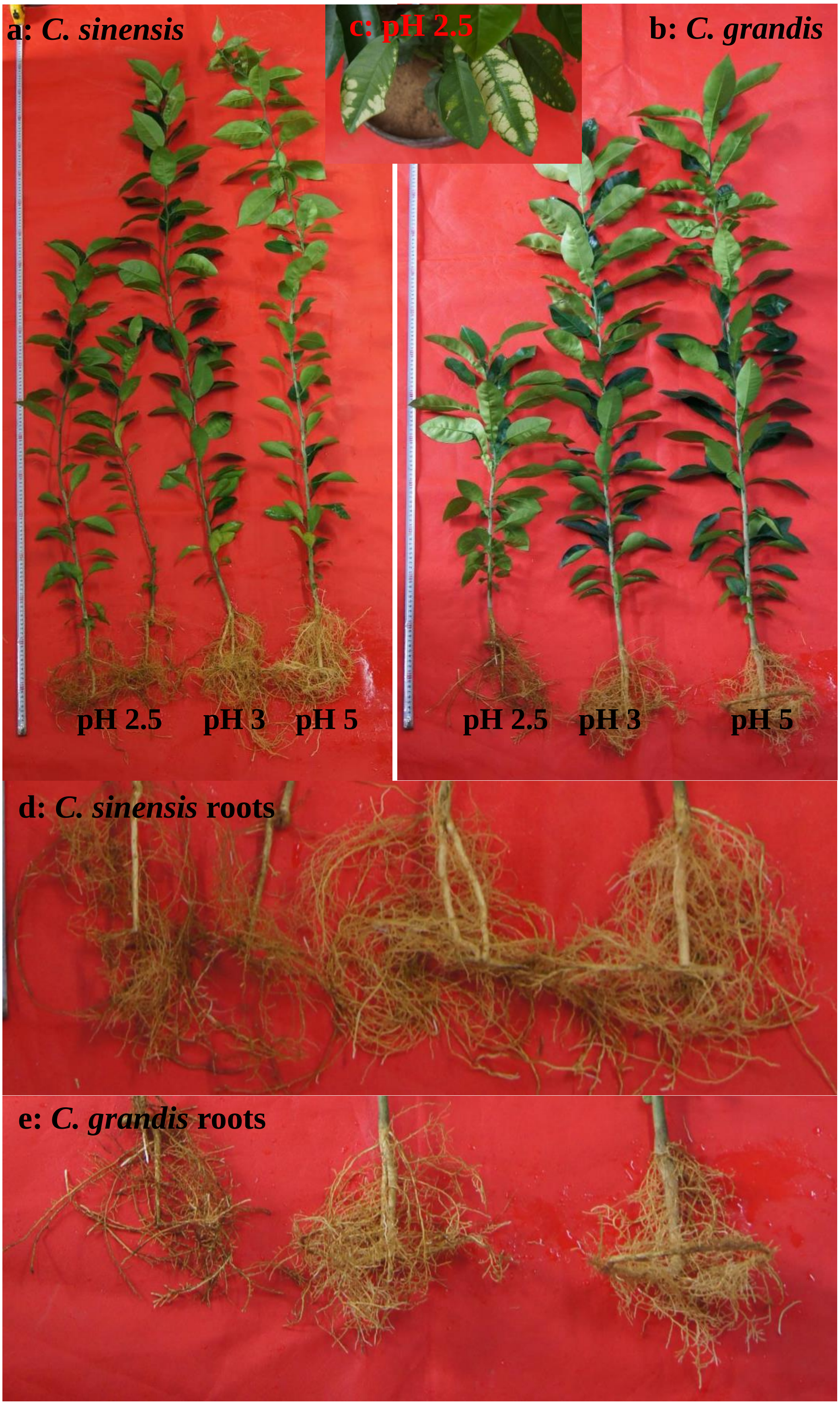

Figure S1. Effects of low pH on *Citrus sinensis* (**a**) and *Citrus grandis* (**b**) seedlings, *C. grandis* leaves (**c**), and *C. sinensis* (**d**) and *C. grandis* (**e**) roots.

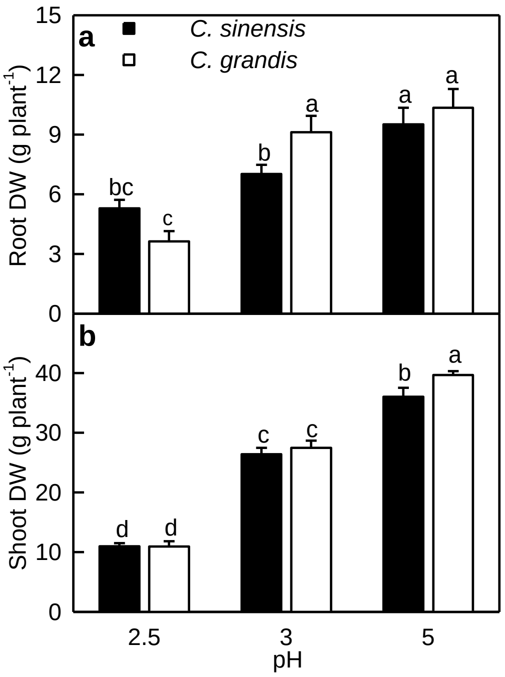

Figure S2. Effects of low pH on root (**a**) and shoot (**b**) dry weight (DW) of *Citrus grandis* and *Citrus sinensis* seedlings. Nine months after the pH treatments began, 10 seedlings per treatment from 10 pots were harvested. The seedlings were divided into roots and shoots (stems + leaves). Their DW was weighted after being dried to a constant weight at 70 ℃. Bar represent mean ± SE (n = 10). Different letters above the bars indicate a significant difference at $P < 0.05$.

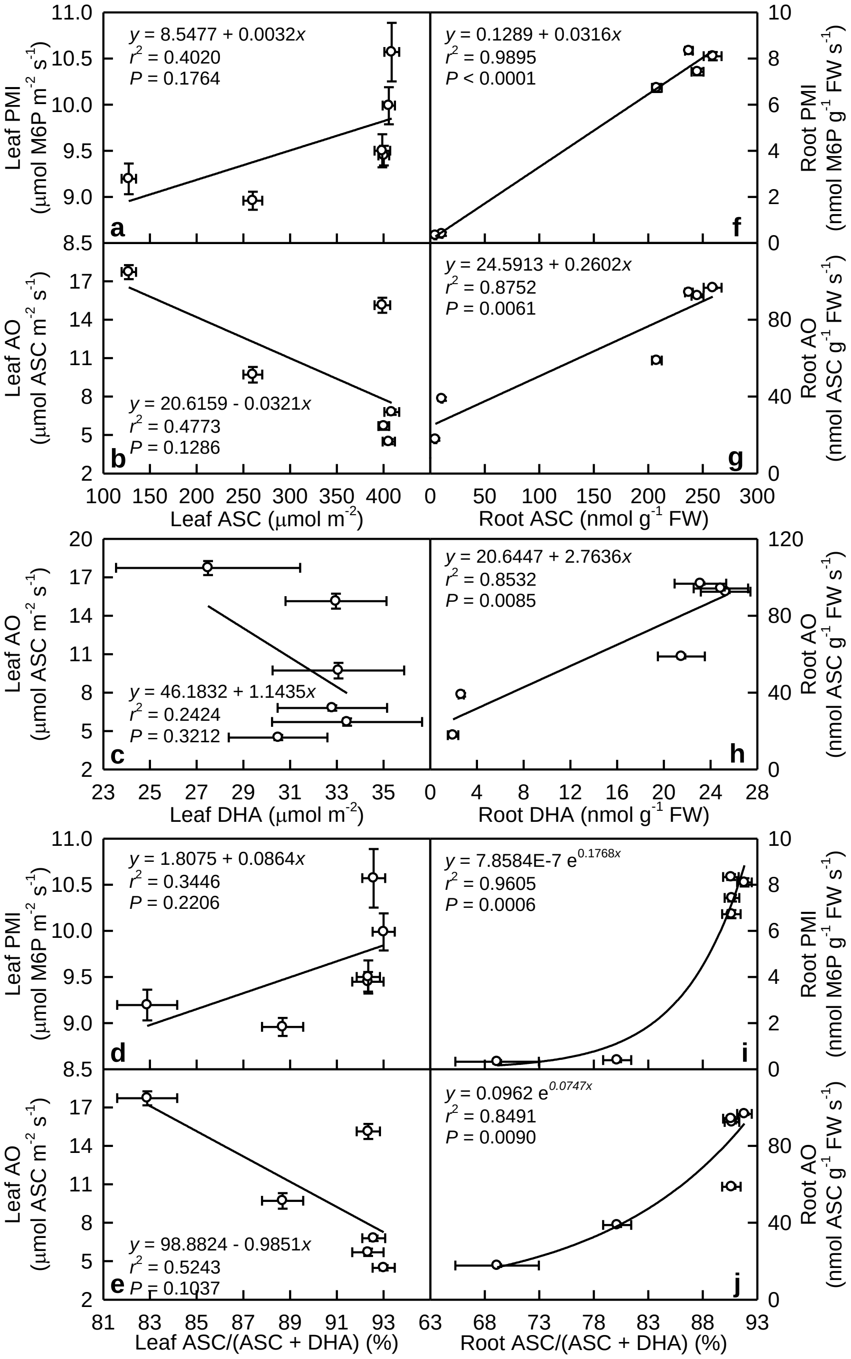

Figure S3. Phosphomannose isomerase (PMI) and ascorbate (ASC) oxidase (AO) activities in relation to ASC and dehydroascorbate (DHA) concentrations and ASC/(ASC + DHA) ratio in leaves (**a-e**) and roots (**f-j**). Points represent mean ± SE for the independent variables (n = 8) and the dependent variables (n = 8).

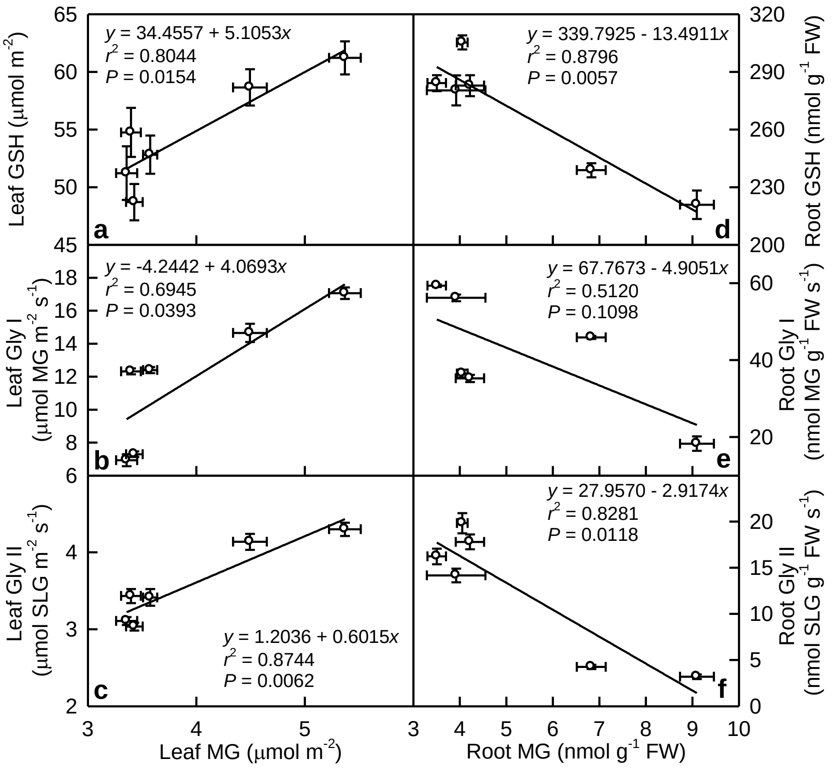

Figure S4. Reduced glutathione (GSH) concentration and glyoxalase (Gly) I and Gly II activities in relation to methylglyoxal (MG) concentration in leaves (**a-c**) and roots (**d-f**). Points represent mean ± SE for the independent variables (n = 4) and the dependent variables (n = 4 except for 8 for GSH).

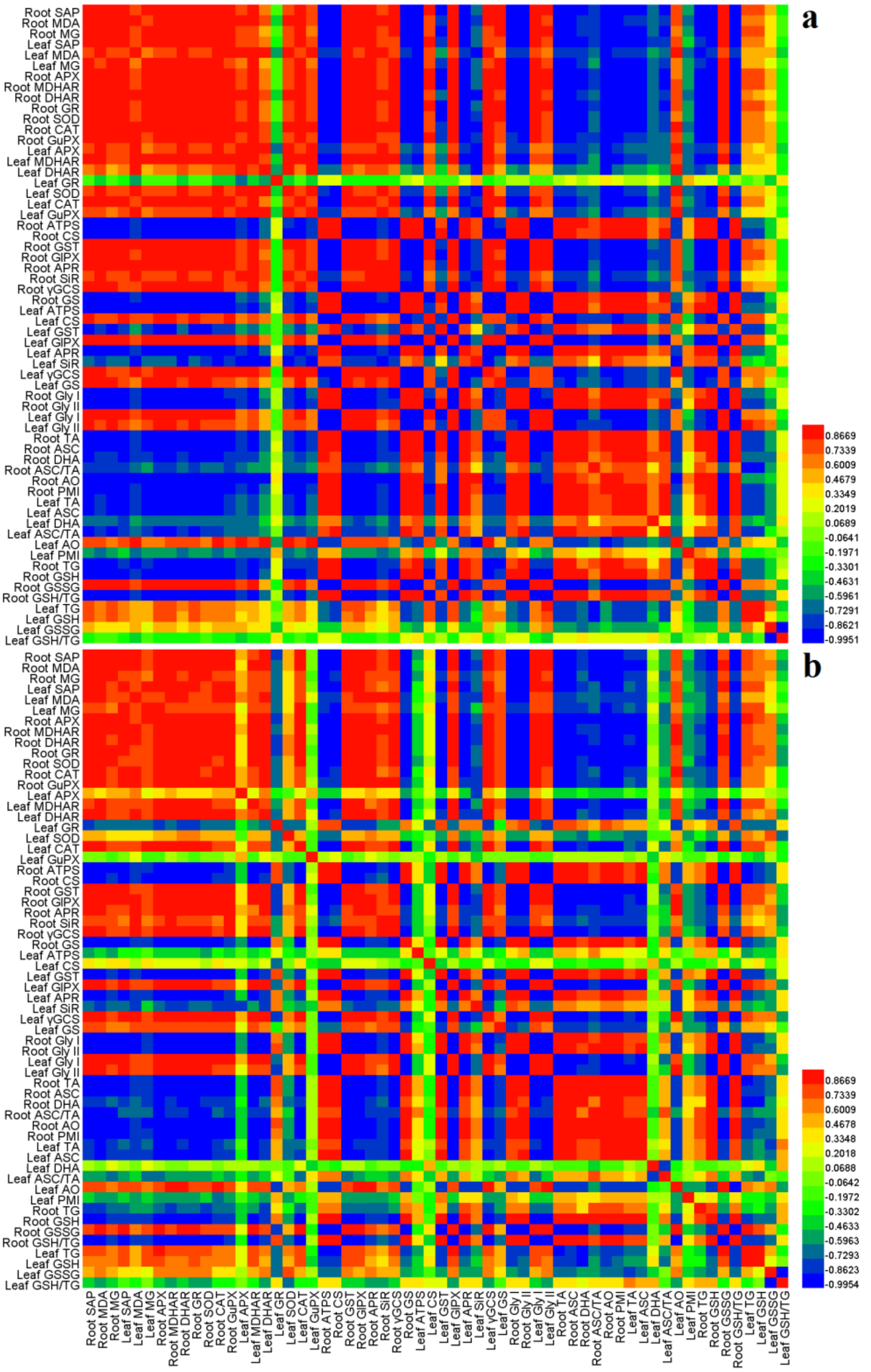

Figure S5. Matrices of Pearson correlation coefficients among the 60 physiological parameters in *Citrus grandis* (**a**) and *Citrus sinensis* (**b**) seedlings. TA: ascorbate (ASC) + dehydroascorbate (DHA); TG: reduced glutathione (GSH) + oxidized glutathione (GSSG).

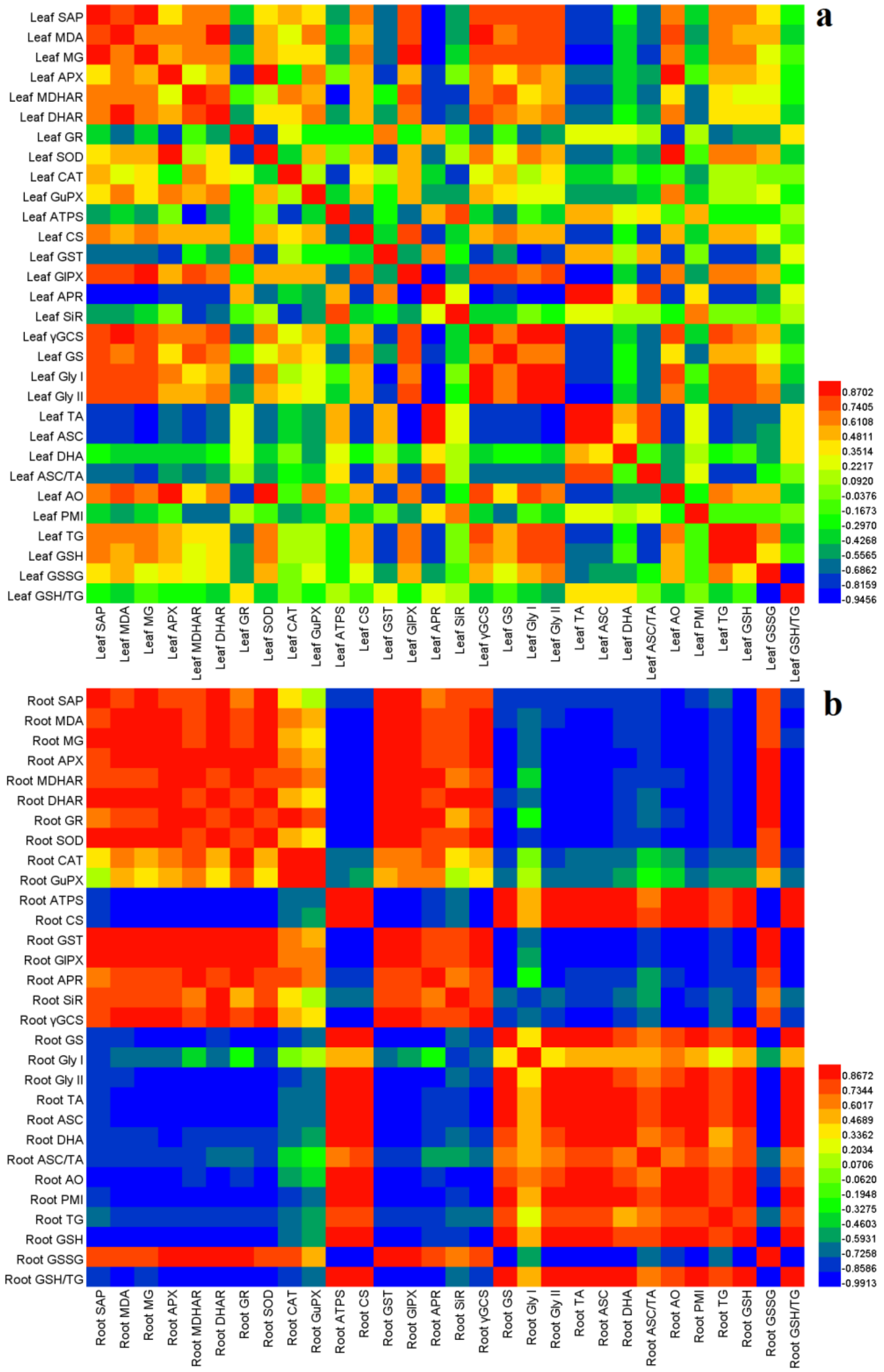

Figure S6. Matrices of Pearson correlation coefficients among the 30 physiological parameters in leaves (**a**) and roots (**b**). TA: ascorbate (ASC) + dehydroascorbate (DHA); TG: reduced glutathione (GSH) + oxidized glutathione (GSSG).

Table S1. Principal component analysis (PCA) for physiological parameters of *Citrus grandis* seedlings

Variables	PC1	PC2	PC3	PC4
**Superoxide anion production, MDA and MG**				
Root superoxide anion production rate	0.9879	-0.1487	0.0039	0.0184
Root MDA	0.9601	0.1097	0.1327	0.1167
Root MG	0.9567	-0.1939	0.1327	0.0471
Leaf superoxide anion production rate	0.9355	-0.0876	0.0138	-0.0742
Leaf MDA	0.8771	0.3856	-0.0631	0.1476
Leaf MG	0.9214	-0.1192	0.2208	-0.2252
**Root antioxidant enzymes**				
Root APX	0.9843	-0.1663	0.0142	0.0205
Root MDHAR	0.9872	-0.1299	0.0030	0.0582
Root DHAR	0.9641	0.1730	0.0130	-0.0664
Root GR	0.9888	-0.1376	-0.0108	-0.0287
Root SOD	0.9684	-0.0937	0.1363	-0.0563
Root CAT	0.9795	0.1429	-0.0273	-0.0137
Root GuPX	0.9371	0.0811	-0.0161	0.0984
**Leaf antioxidant enzymes**				
Leaf APX	0.8492	0.4517	-0.1280	0.0347
Leaf MDHAR	0.9374	0.2745	0.0782	-0.1110
Leaf DHAR	0.7360	0.6194	-0.0113	0.2065
Leaf GR	-0.2675	-0.6974	0.3401	-0.1210
Leaf SOD	0.8868	0.3525	-0.0102	0.1526
Leaf CAT	0.9366	-0.0448	0.1807	-0.0754
Leaf GuPX	0.8232	0.4810	0.0885	0.0140
**Root S metabolism-related enzymes**				
Root ATPS	-0.9774	0.0818	-0.0554	0.0396
Root CS	-0.9861	0.0894	-0.0298	-0.0833
Root GST	0.9954	0.0108	-0.0070	-0.0206
Root GlPX	0.9890	-0.1233	-0.0538	0.0031
Root APR	0.9719	0.0676	0.0647	-0.0834
Root SiR	0.8637	0.3556	0.2144	-0.0965
Root γGCS	0.9310	0.0399	0.0322	-0.1762
Root GS	-0.9672	0.2136	-0.0233	0.0541
**Leaf S metabolism-related enzymes**				
Leaf ATPS	-0.9844	-0.0162	0.0237	0.0808
Leaf CS	0.8932	-0.1135	0.0390	0.1642
Leaf GST	-0.8503	0.4261	-0.0652	0.0329
Leaf GlPX	0.9747	-0.1381	0.0753	0.0025
Leaf APR	-0.9831	0.0371	-0.0656	-0.0100
Leaf SiR	-0.8104	-0.5546	-0.0028	0.0619
Leaf γGCS	0.9497	0.1941	0.0036	0.0533
Leaf GS	0.8738	-0.0954	0.3219	-0.0059
**Root Gly I and Gly II**				
Root Gly I	-0.9479	0.1153	-0.0592	0.2521
Root Gly II	-0.9732	0.1293	-0.0013	0.0011
**Root Gly I and Gly II**				
Leaf Gly I	0.9603	-0.1965	0.0009	-0.1381
Leaf Gly II	0.9086	-0.2065	-0.2031	0.0749

**Root ascorbate and related enzymes**				
Root ASC + DHA	-0.9922	0.0929	0.0350	0.0329
Root ASC	-0.9935	0.0879	0.0177	0.0128
Root DHA	-0.8944	0.1304	0.1901	0.2130
Root ASC/(ASC + DHA)	-0.7702	0.2324	0.2205	-0.5133
Root AO	-0.9586	-0.2597	-0.0636	-0.0248
Root PMI	-0.9963	0.0472	-0.0262	0.0087
**Leaf ascorbate and related enzymes**				
Leaf ASC + DHA	-0.9496	0.2078	0.0173	0.0790
Leaf ASC	-0.9467	0.2125	0.0062	0.0566
Leaf DHA	-0.7109	0.0601	0.2099	0.4525
Leaf ASC/(ASC + DHA)	-0.8526	0.2548	-0.1019	-0.2714
Leaf AO	0.8128	0.4431	-0.1160	0.1081
Leaf PMI	-0.5826	-0.4796	-0.2953	-0.0947
**Root glutathione**				
Root GSH + GSSG	-0.9151	-0.0977	-0.1216	-0.2100
Root GSH	-0.9836	0.0160	-0.0490	0.0908
Root GSSG	0.9583	-0.0977	-0.0594	-0.1758
Root GSH/(GSH + GSSG)	-0.9759	0.1101	0.0178	0.1214
**Leaf glutathione**				
Leaf GSH + GSSG	0.7477	-0.3619	-0.4187	0.3379
Leaf GSH	0.7159	-0.4568	-0.1348	0.4652
Leaf GSSG	0.4943	-0.0046	-0.8434	-0.0783
Leaf GSH/(GSH + GSSG)	-0.3012	-0.1385	0.8715	0.2413
**Eigen value**	48.588	3.898	2.442	1.517
**Variation percent (%)**	80.979	6.497	4.071	2.529

Table S2. Principal component analysis (PCA) for physiological parameters of *Citrus sinensis* seedlings

**Variables**	**PC1**	**PC2**	**PC3**	**PC4**	**PC5**	**PC6**
**Superoxide anion production, MDA and MG**						
Root superoxide anion production rate	0.9747	-0.0213	0.0932	-0.0529	-0.0845	0.0208
Root MDA	0.9867	0.0743	0.0374	-0.1014	-0.0275	0.0081
Root MG	0.8970	-0.1357	0.1259	-0.1314	-0.1784	-0.2078
Leaf superoxide anion production rate	0.9206	-0.1293	0.2020	0.2018	0.0864	0.1522
Leaf MDA	0.8714	0.1824	0.3308	-0.0599	0.1987	-0.0296
Leaf MG	0.8446	-0.2473	0.0461	0.1639	0.2815	-0.1033
**Root antioxidant enzymes**						
Root APX	0.9939	-0.0684	-0.0462	0.0238	0.0198	-0.0176
Root MDHAR	0.9819	0.1403	0.0892	-0.0642	0.0003	-0.0023
Root DHAR	0.9892	0.0514	0.0124	0.0929	0.0108	0.0131
Root GR	0.9963	0.0128	-0.0434	0.0033	0.0000	-0.0201
Root SOD	0.9822	0.0520	-0.0414	-0.1427	-0.0219	0.0163
Root CAT	0.9137	0.0482	0.2166	0.1846	0.1279	-0.1230
Root GuPX	0.9104	0.0404	0.2904	-0.0093	0.1288	-0.0198
**Leaf antioxidant enzymes**						
Leaf APX	0.4417	0.0621	0.7737	-0.0601	-0.1019	0.1891
Leaf MDHAR	0.9133	-0.0730	-0.1750	-0.0981	0.2744	0.0996
Leaf DHAR	0.9204	0.2284	0.2552	-0.0767	0.0117	-0.0936
Leaf GR	-0.7548	-0.0518	0.0260	-0.5077	0.3155	0.1587
Leaf SOD	0.6028	0.2579	-0.4838	0.2027	-0.0744	-0.2593
Leaf CAT	0.9075	-0.2352	-0.0623	-0.0080	-0.0331	0.0517
Leaf GuPX	-0.0529	0.6953	0.5958	-0.1570	0.0756	0.1288
**Root S metabolism-related enzymes**						
Root ATPS	-0.9844	0.0933	0.0414	-0.0676	0.0431	-0.0219
Root CS	-0.9652	0.1406	0.1187	-0.0928	0.0139	0.0076
Root GST	0.9924	-0.0755	-0.0569	0.0158	0.0015	0.0108
Root GlPX	0.9970	-0.0124	0.0537	0.0155	0.0188	-0.0050
Root APR	0.9086	0.2604	0.1742	0.1070	-0.0889	-0.0931
Root SiR	0.8248	0.2130	0.0921	-0.2888	0.0794	-0.2703
Root γGCS	0.9420	0.0619	-0.0316	-0.1739	0.2009	0.0943
Root GS	-0.9743	0.0991	-0.0568	0.0855	0.0108	0.0106
**Leaf S metabolism-related enzymes**						
Leaf ATPS	-0.4305	0.1489	0.1549	0.4821	0.5505	-0.0299
Leaf CS	0.2940	-0.5058	0.3660	0.4768	-0.4308	-0.3129
Leaf GST	-0.9578	0.0901	0.0077	-0.1421	-0.1597	0.0881
Leaf GlPX	0.9746	-0.0137	-0.1303	-0.0205	0.0223	0.0963
Leaf APR	-0.9485	0.0448	0.0478	-0.0094	0.0389	-0.1314
Leaf SiR	-0.7258	-0.3193	-0.4516	-0.1474	0.2203	-0.1437
Leaf γGCS	0.9833	0.1262	0.0372	0.0169	-0.0367	0.0829
Leaf GS	0.7682	-0.0100	-0.0549	-0.2934	0.4937	-0.1521
**Root Gly I and Gly II**						
Root Gly I	-0.9806	0.0374	-0.1355	0.0133	-0.0216	-0.0459
Root Gly II	-0.9744	-0.0038	-0.0519	0.0653	0.0533	-0.0410
**Root Gly I and Gly II**						
Leaf Gly I	0.9766	-0.0825	0.0041	-0.0065	0.0443	0.0965
Leaf Gly II	0.9565	0.0595	-0.1470	0.1116	-0.0868	0.0768

**Root ascorbate and related enzymes**						
Root ASC + DHA	-0.9945	0.0456	0.0310	0.0139	0.0202	-0.0434
Root ASC	-0.9952	0.0405	0.0120	0.0237	0.0372	-0.0263
Root DHA	-0.8935	0.0923	0.2197	-0.0861	-0.1535	-0.2115
Root ASC/(ASC + DHA)	-0.8709	0.1647	-0.0209	0.3468	0.1904	0.1568
Root AO	-0.9938	0.0458	0.0078	0.0084	-0.0080	-0.0274
Root PMI	-0.9916	0.0150	0.0020	0.0411	0.0217	-0.0050
**Leaf ascorbate and related enzymes**						
Leaf ASC + DHA	-0.8802	-0.2437	0.2529	0.1500	-0.1980	0.0720
Leaf ASC	-0.9059	-0.1268	0.2303	0.1854	-0.1848	0.0902
Leaf DHA	0.1006	-0.8858	0.1924	-0.2452	-0.1171	-0.1264
Leaf ASC/(ASC + DHA)	-0.5686	0.7017	-0.0584	0.3463	0.0028	0.1227
Leaf AO	0.8681	0.2998	0.0650	0.2762	0.0831	0.1751
Leaf PMI	-0.5207	-0.3004	-0.0285	-0.1961	-0.1960	0.6808
**Root glutathione**						
Root GSH + GSSG	-0.7166	-0.0853	-0.1610	0.2704	0.3766	0.0134
Root GSH	-0.9555	0.0424	0.1429	0.2158	0.0540	-0.0771
Root GSSG	0.9185	-0.0997	-0.0894	0.3139	0.0207	0.1356
Root GSH/(GSH + GSSG)	-0.9483	0.0489	0.0759	-0.2164	0.0555	-0.0847
**Leaf glutathione**	0.8449	-0.2291	-0.3017	0.1662	-0.0791	0.1561
Leaf GSH + GSSG	0.7525	-0.4708	-0.1990	0.2140	0.0406	0.1764
Leaf GSH	0.6394	0.5202	-0.4096	-0.0455	-0.3485	0.0211
Leaf GSSG	-0.4375	-0.7048	0.3559	0.1168	0.3822	0.0419
Leaf GSH/(GSH + GSSG)						
**Eigen value**	44.870	4.055	2.802	2.099	1.895	1.256
**Variation percent (%)**	74.784	6.759	4.670	3.499	3.159	2.093

Table S3. Principal component analysis (PCA) for physiological parameters of leaves and roots

Variables	Leaves					Roots	
	PC1	PC2	PC3	PC4	PC5	PC1	PC2
**Superoxide anion production, MDA and MG**							
Superoxide anion production rate	0.8750	-0.1877	0.1742	-0.0272	0.1438	0.8774	-0.3985
MG	0.8846	-0.1439	0.2324	0.0797	-0.0485	0.9291	-0.2069
MDA	0.8909	-0.0227	-0.1609	0.2006	0.2160	0.9560	-0.1328
**Antioxidant enzymes**							
APX	0.7121	0.4887	-0.2193	0.3413	-0.1448	0.9786	-0.0627
CAT	0.3660	-0.8242	0.1717	-0.1977	0.2298	0.7209	0.6432
SOD	0.6947	0.5889	-0.1216	0.2315	-0.1617	0.9488	-0.2457
MDHAR	0.7505	-0.5919	-0.0891	0.0276	0.0453	0.9469	0.2260
GuPX	0.5813	-0.1084	-0.5345	0.4313	-0.2466	0.5905	0.7225
GR	-0.5563	-0.6068	0.2422	-0.1158	-0.3099	0.9234	0.3538
DHAR	0.7923	-0.2086	-0.2917	0.2070	0.3713	0.9414	-0.1755
**S metabolism-related enzymes**							
GS	0.7899	-0.3515	0.2102	0.1119	-0.0230	-0.9482	-0.1956
GST	-0.7094	-0.5695	-0.2157	-0.0337	-0.2409	0.9881	-0.0789
SiR	-0.4032	0.7895	0.3331	-0.0599	-0.1649	0.7781	-0.4135
CS	0.7510	-0.2014	0.1112	0.1058	-0.3745	-0.9768	0.0252
γGCS	0.9315	0.1393	-0.0293	0.0110	0.2494	0.9236	-0.1699
ATPS	-0.5143	0.7697	0.0558	0.1554	0.1885	-0.9738	-0.0269
APR	-0.9647	-0.0145	-0.0936	-0.0092	0.0345	0.8781	0.3084
GlPX	0.9346	-0.2119	0.1227	-0.0505	-0.1812	0.9933	0.0061
**Gly I and II**							
Gly II	0.9090	0.0852	0.1084	-0.2859	0.1688	-0.9486	-0.2026
Gly I	0.8656	0.3597	0.1858	-0.0381	0.1677	-0.5716	0.7709
**Ascorbate and related enzymes**							
DHA	-0.4175	-0.0441	0.5453	0.1806	0.4284	-0.8876	-0.0198
ASC/(ASC + DHA)	-0.7602	0.0409	-0.4497	-0.0878	0.1651	-0.7707	0.1750
ASC + DHA	-0.9170	0.0748	-0.0551	0.1071	0.2837	-0.9825	-0.0062
ASC	-0.9192	0.0808	-0.0978	0.0975	0.2620	-0.9830	-0.0048
PMI	-0.4405	0.5014	0.2230	-0.2359	-0.2922	-0.9893	-0.0707
AO	0.8330	0.3494	-0.2414	0.2292	-0.0302	-0.9402	0.2177
**Glutathione**							
GSSG	0.6112	0.2458	-0.3849	-0.6127	0.0292	0.9406	0.0950
GSH/(GSH + GSSG)	-0.4049	-0.1775	0.5831	0.6347	-0.0596	-0.9645	-0.0963
GSH + GSSG	0.8118	0.3066	0.2458	-0.3017	-0.0011	-0.8122	-0.2063
GSH	0.7543	0.2788	0.4593	-0.1241	-0.0132	-0.9725	0.0717
**Eigen value**	16.7606	4.6533	2.3078	1.6922	1.3314	24.7262	2.5519
**Variation percent (%)**	55.8687	15.5110	7.6926	5.6408	4.4380	82.4205	8.5063
